# National insights on malignant hyperthermia: a SIAARTI survey on clinical practices, preparedness, and future directions

**DOI:** 10.1186/s44158-025-00293-4

**Published:** 2025-11-19

**Authors:** Roberta Monzani, Daniela Alampi, Elena Bignami, Andrea Cortegiani, Antonino Giarratano, Fabrizio Racca, Fabio Sbaraglia

**Affiliations:** 1 Department of Anesthesia and Intensive Care, Humanitas Research Hospital, Rozzano (MI), Fondazione Policlinico Universitario A. Gemelli IRCCS, Università Cattolica del Sacro Cuore, Largo F. Vito 1, Rome, MI 00135 Italy; 2 Sapienza University of Rome, A.O.U. Sant’Andrea, Rome, Italy; 3 Intensive Care and Pain Medicine Division, Department of Medicine and Surgery, University of Parma, Parma, 43126 Italy; 4 Department of Precision Medicine in Medical, Surgical and Critical Care (Me.Pre.C.C.), University of Palermo, Palermo, Italy; 5 Department of Anesthesia Intensive Care and Emergency, University Hospital Policlinico Paolo Giaccone, Palermo, Italy; 6 Division of Anesthesia and Critical Care Medicine, AO Ordine Mauriziano, Turin, 10128 Italy; 7 Department of Anesthesia and Intensive Care, Fondazione Policlinico Universitario A. Gemelli IRCCS, Rome, 00168 Italy

**Keywords:** Malignant hyperthermia, Anesthesia, Dantrolene, Adverse events

## Abstract

**Background:**

Malignant hyperthermia (MH) syndrome is a rare pharmacogenetic disorder that can be highly life-threatening if diagnosis and treatment are delayed. The purpose of this study is to assess the knowledge and current practices of Italian anesthesiogists in managing malignant hyperthermia episodes.

**Methods:**

We conducted a national survey. Data were collected via an online questionnaire distributed by the Italian Society of Anaesthesia, Analgesia, Resuscitation and Intensive Care (SIAARTI). Responses were collected over 15 weeks between July 15 and October 15, 2024, using an online General Data Protection Regulation-compliant platform.

**Results:**

A total of 395 anesthetists completed the survey. The majority are employed in public (35%) and university hospitals (26%), with an average of 20 years of professional experience. MH had been managed at least once by 31% of respondents, and 70% of them declared they always report adverse reactions.

In over 90% of cases, preventive measures (removal of trigger drugs, ventilator wash-out, perioperative care) are identified, although only 49% reported having an internal protocol in place at their institution.

In most centers (89%), non-anesthesiologists are responsible for the storage and supply of dantrolene and only 66% of respondents correctly identify sterile water as its appropriate solvent.

**Discussion:**

Our results highlight the need for broader standardization of MH management. Despite limitation in sample size and difference in geographical and hospital setting, the survey reveals a discrepancy between clinical practice and recommended strategies. Although the need for preventive measures as a mean to avoid episodes of MH is widely recognized, there continues to be too much ambiguity on what the exact protocol should be in these situations, leaving room to develop an unequivocal approach that allows the optimal treatment for episodes of MH.

## Background

Malignant hyperthermia (MH) is a rare but life-threatening pharmacogenetic disorder affecting skeletal muscle metabolism, primarily triggered by exposure to volatile anesthetics and depolarizing muscle relaxants [1]. The use of these drugs in susceptible subjects can cause an uncontrolled calcium release from the sarcoplasmic reticulum due to RYR1 gene mutations. It generally presents with muscle rigidity, hypercapnia, tachycardia, hyperthermia, and rhabdomyolysis. If not promptly diagnosed and treated, MH can rapidly lead to multi-organ failure and death [2]. Immediate treatment includes discontinuing triggering agents, administering intravenous dantrolene, active cooling, correcting acidosis, managing hyperkalemia, and supporting vital functions to prevent multi-organ failure. National societies tried to define preventive and treatment guideline over the years, reaching the goal of drastically reduced mortality to less than 5% [3]. However, misconceptions, overtreatment, or imprecise diagnostic pathway are still rather common [4].

Given its critical clinical significance, SIAARTI conducted a nationwide survey among Italian anesthesiologists and critical care specialists to assess their experience, knowledge, and preparedness in managing MH cases. The findings of the survey were integrated with expert opinions to provide an in-depth analysis of current clinical practices, challenges, and recommended strategies.

## Methods

### Setting, participant and recruitment

This study is based on a nationwide online survey developed by experts belonging to SIAARTI (the Italian Society of Anaesthesia, Analgesia, Resuscitation, and Intensive Care) and approved by its research committee approval in 2024. IRB approval was not required for this study. The survey aimed to assess the knowledge of healthcare professionals regarding MH, with a particular focus on clinical management strategies and the use of dantrolene. Given the rarity of the phenomenon under investigation and the lack of sufficient preliminary data to estimate key parameters, such as response rate or expected variability, it was not feasible to define a reference sample size. Convenience sampling and a descriptive study design were therefore deemed the most appropriate methodological approach for the analysis of this rare population [5].

Data collection was conducted between July 15 and October 15, 2024, through an online General Data Protection Regulation-compliant platform (SurveyMonkey 2024, Survey tool, Momentive Inc.), ensuring anonymity while allowing robust data aggregation and analysis.

The survey was delivered by mail to among 11,000 anesthesiologists and intensivists across Italy via SIAARTI’s network, targeting professionals with direct involvement in perioperative care and intensive care management. Before accessing the survey, each participant has given their consent to take part in the study.

### Questionnaire design

The survey was structured into three main sections designed to gather a comprehensive understanding of how MH is managed in clinical settings:

1. Demographic and professional data
  o Age distribution and professional background.
  o Geographic location (region and city of practice) to evaluate potential regional disparities.
  o Year of specialization to define levels of seniority and professional expertise.
  o Type of healthcare facility (public, private, university hospital, or other institutions) to determine differences in resource availability and adherence to standardized protocols, in cases where they were already established.
2. Experience with malignant hyperthermia
  o Number of MH cases managed during the years as active professional or training programs.
  o Identification of patient groups perceived as at risk for MH, including those with neuromuscular disorders or a history of adverse anesthetic reactions.
  o Differential diagnosis awareness, including conditions that can mimic MH, such as neuroleptic malignant syndrome, sepsis, or thyroid storm (thyrotoxic crisis)
3. Management strategies
  o Existence of codified hospital protocols for MH crisis management.
  o Preventive measures taken to mitigate the risk of MH, including preoperative screening and selection of non-trigger anesthetics.
  o Use of dantrolene in preoperative prophylaxis and the extent to which institutions maintain an adequate stock.
  o Post-crisis patient management, including referral to specialized centers and pharmacovigilance reporting practices.

Free comment fields were available to elicit further information where appropriate. The estimated time to complete the survey was 20 min. Each survey was reviewed for readability and non-ambiguity by experts in MH.

### Statistical analysis

The collected data were analyzed using descriptive statistical methods, including frequency distributions, medians, means, standard deviations, and other summary statistics to characterize the key variables. Qualitative variables were examined using contingency tables and graphical representations to provide a clear visualization of response distributions.

For ordinal scale variables, such as the frequency of drug use or the application of dantrolene prophylaxis, an agreement indicator was employed. This indicator synthesizes response convergence: values close to 1 reflect consensus regarding the adoption of specific practices; values closer to 0 reflect divergence in clinical decision-making. This approach was used to provide a structured measurement of consensus levels on complex clinical decisions.

## Results

### Characteristics of the sample

The total number of professionals who responded to the survey was 395.

The average age of the sample was 46 years (26 ÷ 76 years) and the age group 31 ÷ 50 gave 61% of the answers.

**Fig. 1:**
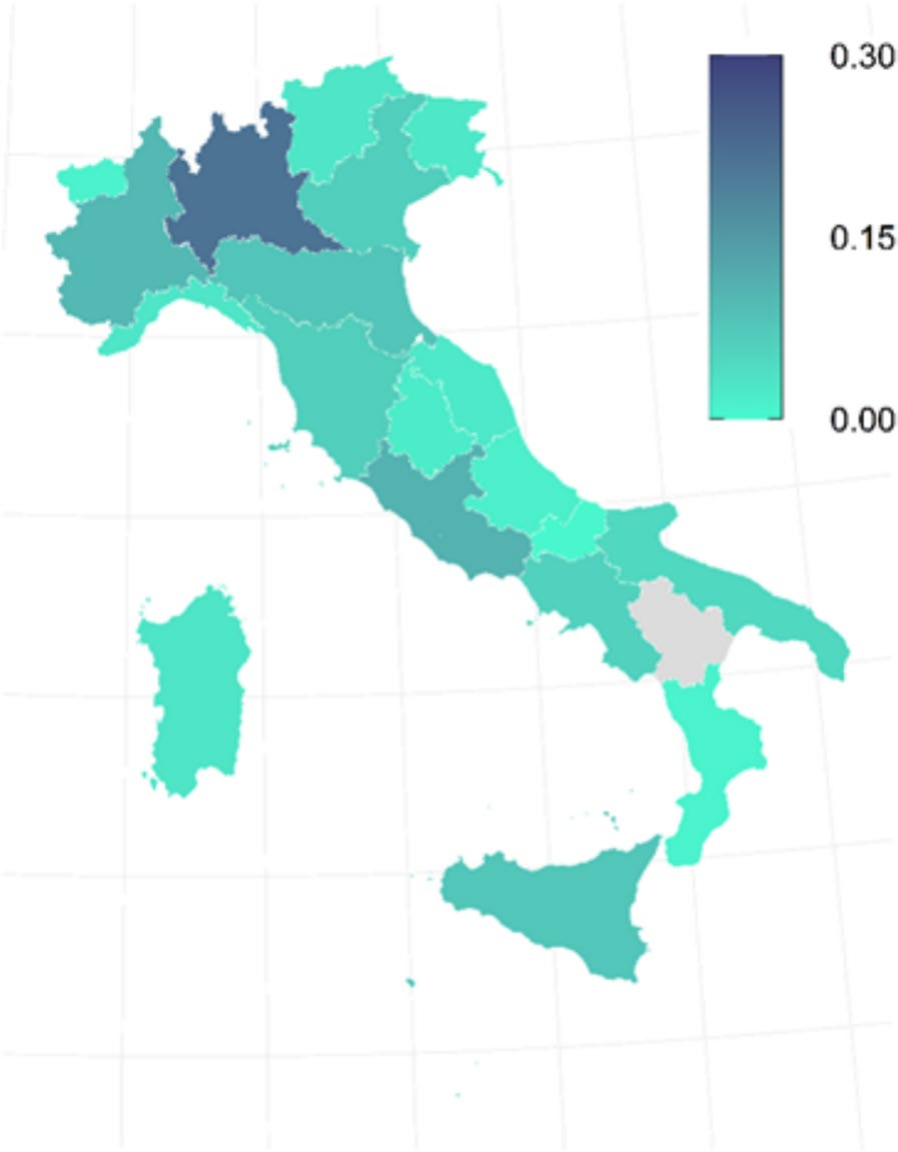
Sample geographical distribution rate. 0.1 is 10% of respondents

Geographically, the sample was mainly composed of professionals from Northern Italy (55%), followed by Central (24%) and Southern Italy (21%). Taking into account the number of SIAARTI members surveyed, the regional distribution ranges from a higher concentration of responses from Lombardia (22%), followed by Lazio (11%) and Emilia-Romagna (8%), until Basilicata (0%) (Fig. 1).

The average length of work experience is 20 years with a standard deviation of 12 years.

The majority of them are employed in public hospitals (35%) and university hospitals (26%).

### Management of malignant hyperthermia

The survey provided valuable insights into how MH is managed in Italian healthcare facilities, from prevention strategies to crisis response and post-crisis care. Institutional preparedness for MH varies significantly across healthcare settings (Table 1).

**Table 1:** Case distribution for institutional setting

MH patients treated	Nr (%)
Major Public Hospital	41 (35%)
University Hospital	30 (26%)
Minor Public Hospital	15 (13%)
Private IRCCS	5 (4%)
Public IRCCS	7 (6%)
Private Hospital with public role	11 (9%)
Private Hospital	5 (4%)
Other	2 (2%)
**Total**	**116 (100%)**

Almost a third of the professionals surveyed (31%, *n* = 116) have stated having experienced an occurrence of MH, while only 7% have experience more than 5 cases during their practice. There is a geographical trend with higher incidence in Central Italy (50%) and in public (35%) and university (26%) hospitals. The recurrence of MH in patients previously exposed to trigger agents showed a reported prevalence of 1 to 15%, where approximately 50% of MH cases occur in patients with previous uneventful exposures.

A consistent trend emerged in the adoption of preventive measures (Table 2), with 91% of respondents confirming that they actively avoid the use of volatile anesthetics and succinylcholine in patients identified as being at risk.

**Table 2:** Preventive strategies—age group

Age group	I avoid exposure in subjects susceptible to trigger drugs	I identify susceptible subjects	I identify susceptible subjects and avoid exposure to trigger drugs	I do not implement strategies	Other
< 30	0 (0%)	1 (5%)	18 (95%)	0 (0%)	0 (0%)
31–40	6 (5%)	1 (1%)	119 (92%)	3 (2%)	1 (1%)
41–50	8 (6%)	1 (1%)	130 (92%)	0 (0%)	1 (1%)
51–60	6 (7%)	0 (0%)	80 (92%)	1 (1%)	0 (0%)
> 61	6 (10%)	0 (0%)	53 (87%)	0 (0%)	2 (3%)
**Total**	**26 (6%)**	**3 (1%)**	**400 (91%)**	**4 (1%)**	**4 (1%)**

The use of trigger agents was found to be uniformly low, with a Likert-scale indicator of 0.07 confirming that these drugs are almost never administered to susceptible patients.

A disparity was found in testing for creatine phosphokinase (CK) haematic level in preoperative setting: 51% of participants surveyed only test for CK in high-risk patients, while 38% require the test regardless of patient conditions.

The survey highlights that, while 49% of respondents stated that their healthcare facilities provide an internal protocol for the management of MH, 21% have declared that their facility, on the contrary, does not provide such a protocol. Furthermore, 16% of professionals surveyed have stated that they are not aware of any specific documentation on MH.

### Intraoperative management

The preventive measures employed to prepare the mechanical ventilator are standardized and homogenous across the sample analyzed: the removal of the vaporizer, the changing of the soda lime canister and the replacement of the ventilation circuits are widely recognized as part of the optimal preparation for patients at risk of MH [6]. However, in the case of pregnant patients requiring non-obstetric surgery, 71% of respondents favored total intravenous anesthesia (TIVA) over neuraxial anesthesia (25%).

Another issue where a clear consensus was not reached is the correct way to prepare dantrolene, with only 66% of respondents identifying sterile water as the appropriate solvent for dantrolene dilution [7] (Table 3).

**Table 3:** Diluent indicated for dantrolene reconstitution

Diluent	Nr (%)
NaCl 0,9%	86 (20%)
Glucose 5%	46 (11%)
Water for injectable preparation	276 (66%)
Bicarbonate	2 (1%)
Other	10 (2%)

Regarding dantrolene stock management [8], 64% of respondents indicate that the operating room nurse coordinator was responsible for verifying and maintaining dantrolene supply, followed by intensive care nurse coordinators (16%) and hospital pharmacists (9%).

### Postoperative management and discharge

The referral of post-MH patients to specialized centers is reported in 60% of cases; 22% turn to geneticists, 7% to neurologists. However, there is no data regarding how many patients ultimately undergo confirmatory diagnostic testing.

The 59% of respondents report providing both verbal and written discharge instructions about follow-up; 13% give only verbal instructions and 22% inform family members of the risk.

About pharmacovigilance, while 70% of respondents say they always report adverse reactions, 16% do not consider MH an adverse drug reaction, and 23% report accidents only to department heads.

## Discussion

Our results highlight the need for broader standardization and spreading of MH management guidelines across different hospital settings, ensuring that all surgical centers operate under a unified crisis response framework. Despite a limited number of respondents, some interesting results emerge from data analysis.

The main group represented (31–50 years, with an average experience of 20 years) suggests that professional experience correlates with confidence in managing MH emergencies. The standard deviation of 12 years in professional experience could ensure a good balance in the sample detected by the survey.

The low incidence of MH could be the reason why younger practitioners, possibly less experienced in recognizing the clinical pattern of MH, might miss this crucial diagnosis.

The geographical distribution shows potential disparities in MH preparedness. Minor regions are underrepresented with Basilicata completely excluded by the survey. This finding suggests a potential need for further engagement in MH training and protocol implementation in specific regions.

Bigger institutions, where structured protocols and institutional directives are more common, are more represented in the sample. SIAARTI membership could represent a bias due of major engagement of university correlates with society activity. Alternatively, it could be interpreted as that larger institutions may be better equipped to diagnose and manage MH.

Regional differences also emerged in MH case distribution. This geographical variation could be attributed to response rate differences rather than an actual discrepancy in case prevalence. Furthermore, the number of MH cases reported by respondents seems influenced by chance, as MH is not an endemic condition and should theoretically be uniformly distributed across the country.

Data analysis reports that 1 of 3 respondents declared almost an episode of MH. This data could be surprising with respect to the incidence described in literature [9]. However, we have to take into account that response rate is not adequate to validate a standard population and that each MH episode was generally treated by more professional.

Preventive measure adopted in case of susceptible patient suggests a strong adherence to internationally recognized best practices in minimizing MH triggers [10].

Regarding at risk patients, 72% of respondents correctly identified individuals carrying mutations associated with MH and congenital myopathies [11]. Additionally, 91% reported avoiding exposure to trigger agents as a preventive strategy.

International guidelines recommend the absolute avoidance of these drugs in myopathy patients due to the risk of rhabdomyolysis, reinforcing the need for continued training on this issue [12, 13].

However, age-based differences in trigger agent use were noted, with senior professionals demonstrating a lower likelihood of administering these drugs (index of 0.02 vs the overall average of 0.07). This finding suggests that experience may promote a more tailored and cautious approach to anesthesia management.

Geographically, agreement on the use of trigger agents was highest in Calabria (0.29), Umbria (0.14), and Veneto (0.14), which could indicate regional gaps in protocol standardization or disparities in MH training. Addressing these inconsistencies through targeted educational initiatives and internal clinical protocols standardization could help ensure consistent best practices nationwide.

The survey also explored the role of preoperative CK testing [14]. Most respondents agreed with a selective approach (“depends”), aligning with current guidelines recommending CK testing only in cases with a suggestive medical history. However, systematic CK testing was notably more prevalent in public IRCCS facilities (52%), highlighting the need for harmonized preoperative screening protocols.

Data about the use of regional anesthesia, rather than general anesthesia, in patients at risk for MH, is comforting, demonstrating strong adherence to national best practices [6]. On the contrary, the recommend neuraxial anesthesia in case of obstetric anesthesia [15] is not fully considered. This deviation could be explained by factors including a limited access to specialized obstetric anesthesia teams or regional differences in epidural anesthesia utilization.

The necessity of preoperative dantrolene prophylaxis was generally dismissed, aligning with international best practices. However, a pressing issue arose in the reconstitution of the drug, with only 66 of respondents answering correctly. Thus, standardizing dantrolene administration, particularly with the introduction of new formulation [16], remains a key recommendation to ensure optimal preparedness.

Given recent European guideline updates recommending at least 600 mg of dantrolene in each operating theater and at least 1200 mg available hospital-wide, facilities must reassess their storage and inventory strategies [12]. The coexistence of new and old formulation for the next years further necessitates clarity in dosing recommendations, shifting from a vial-based count to precise milligram-based storage guidelines.

Training remains a crucial component of MH preparedness [17]. Simulation-based emergency training should be routinely implemented across all surgical centers, ensuring that operating room personnel are proficient in MH recognition and rapid dantrolene administration. Additionally, updating treatment guidelines to incorporate newer formulations is necessary to optimize response times in crisis scenarios.

A significant concern is pharmacovigilance compliance. Given that European regulations mandate the reporting of all suspected adverse reactions through the EudraVigilance network, increasing compliance with these regulations is critical. The fear of professional or legal repercussions may contribute to underreporting, underscoring the need for greater awareness and simplification of reporting procedures.

Furthermore, improving patient education and discharge protocols is essential [18]. Standardized discharge instructions should provide clear recommendations for future anesthesia procedures, referral pathways, and genetic screening options.

Finally, improving patient education post-MH episode is pivotal [19, 20]. The survey highlighted a lack of standardized discharge criteria, raising concerns about inconsistent patient follow-up. Strengthening the referral process through a standardized national pathway would ensure that at-risk individuals receive appropriate follow-up care. Limited collaboration between anesthesiologists, myologists, and neurologists, as well as the lack of well-publicized specialized centers, contributes to post-discharge challenges.

The development of a nationally recognized patient information protocol, including a formal discharge format and structured follow-up recommendations, is strongly recommended.

The findings of this study emphasize the importance of a structured and interdisciplinary approach to malignant hyperthermia (MH) management. Collaboration among anesthesiologists, pharmacists, myologists, and neurologists is essential to refining diagnostic criteria, improving adherence to standardized protocols, and ensuring effective patient follow-up [21]. Establishing a national communication network among these specialists would facilitate the exchange of knowledge and streamline referral pathways, ultimately enhancing patient care [22].

Ensuring that all operating room personnel are trained to rapidly recognize and manage an MH episode is critical. The survey highlights the necessity of routine simulation-based training and emergency preparedness drills, which should be reinforced across all surgical centers. Additionally, maintaining an adequate and readily accessible stock of dantrolene remains a priority to ensure timely intervention in an MH crisis.

The introduction of new formulation for an update of treatment guidelines regarding dantrolene administration. Clear, standardized recommendations on dosing, preparation, and the specific characteristics of each formulation should be developed to optimize clinical decision-making and reduce delays in treatment. This revision will help ensure that anesthesiologists and emergency teams are equipped with the necessary knowledge to administer the most appropriate formulation in a timely manner.

Pharmacovigilance also remains an area for improvement [23]. Despite European regulations mandating the reporting of adverse drug reactions, MH-related cases appear to be significantly underreported. Encouraging compliance with reporting requirements and simplifying the process would improve epidemiological data, enhance clinical decision-making, and contribute to better public health outcomes.

Addressing these challenges requires a coordinated effort between healthcare professionals, scientific societies, and policymakers. By refining MH management protocols, improving training and preparedness, updating pharmacological guidelines, and enhancing patient referral and pharmacovigilance practices, it is possible to create a more standardized, evidence-based approach to MH care in Italy. Ensuring that at-risk patients receive optimal medical support and structured follow-up care will ultimately lead to improved patient safety and better long-term outcomes in the management of this rare but critical condition.

Overall, the survey results indicate that while strong preventive measures are widely implemented, gaps persist in institutional preparedness, postoperative management, and referral pathways. Addressing these discrepancies through standardization, education, and improved interdisciplinary collaboration will be essential to optimizing MH care in Italy**.**

## Conclusion

This survey underscores the importance of standardizing malignant hyperthermia (MH) management protocols and strengthening interdisciplinary collaboration. Despite widespread adherence to preventive strategies, gaps persist in intraoperative practices, postoperative follow-up, and pharmacovigilance. A unified national approach is essential to ensure timely treatment and long-term care for MH-susceptible patients.

## Limitations

This survey has several limitations. First, when compared to the whole population of anesthesiologists in Italy, the sample size was relatively low to achieve a clear data generalization. In addition, we considered only SIAARTI members, and it could hide a further bias in the sample. Nevertheless, geographical distribution, age categories, and hospital type are respondent with previous national surveys [24]. The last limitation is due to the nature of the survey. Respondents declared their point of view, but, as suggested by pharmacovigilance data, a mismatch with real data could be considered.

## Data Availability

Data is available from the corresponding author upon request.

## Abbreviations

CK: Creatine phosphokinase
EMA: European Medicine Agency
SIAARTI: Italian Society of Anaesthesia, Analgesia, Resuscitation, and Intensive Care
MH: Malignant hyperthermia
TIVA: Total intravenous anesthesia
